# Ophthalmic Disorders in Adult Lymphoma Patients

**DOI:** 10.4103/0974-9233.71582

**Published:** 2010

**Authors:** Mohammad Javed Ali, Santosh Honavar

**Affiliations:** Department of Ocular Oncology, L.V. Prasad Eye Institute, Hyderabad, India

Sir,

We read with great interest the article by Omoti *et al*.^1^ on the spectrum of ophthalmic disorders in adult patients with lymphoma. The article has systematically covered a wide variety of ophthalmic disorders.

However, we do not completely agree with the authors’ contention that most reports of ocular involvement in lymphoma involve few patients. Cassoux *et al*.^2^ reported 42 cases and Hormigo *et al*.^3^ reported 31 cases of primary intraocular lymphoma in their respective study cohorts. Similarly, Shields *et al*.^4^ and Coupland *et al*.^5^ reported 117 and 37 cases, respectively, of conjunctival involvement in lymphoma.

The authors have comprehensively documented the ocular and adnexal involvement in a series of patients with systemic lymphomas. It would also be of interest to the ophthalmologists to look at it from the other point of view. Shields *et al*.^4^ analyzed a large series of conjunctival lymphomas and found that 31% of them are associated with systemic lymphomas. Demirci and coworkers 6,7 in a large series of 160 patients noted that 25% had systemic association with orbital lymphomas, and more interestingly documented that 33% of orbital lymphomas at presentation subsequently developed systemic lymphomas over 10 years.

Omoti *et al*.^1^ excluded all the patients on treatment in their analysis. It could have been possible to still elucidate ocular findings in at least some of these patients which would have further contributed to our understanding of lymphomas.

The article also presents three cases of presumed lymphoma-associated retinopathy based only on features of hemorrhages and vasculitis. Although possible, we believe that angiocentric lymphomas are extremely rare and the classic presentation would be in the form of confluent, round, or geographic greasy-yellow subretinal pigment epithelium infiltrates. 8
